# Complex Reimplantation of Aneurysmal Left Main Coronary Artery During Reoperative Aortic Root Replacement

**DOI:** 10.7759/cureus.93368

**Published:** 2025-09-27

**Authors:** Brandon Sloan, Kaitlin Nguyen, Sean Hamlin, Sanford Zeigler

**Affiliations:** 1 Department of General Surgery, St. Luke's University Health Network, Bethlehem, USA; 2 Department of Surgery, Division of Cardiothoracic Surgery, Medical University of South Carolina, Charleston, USA

**Keywords:** aortic root replacement, aortic surgery, congenital heart disease, coronary artery aneurysm, coronary artery fistula, coronary reimplantation, redo sternotomy

## Abstract

Coronary artery fistula (CAF), an aberrant connection between a coronary artery and a chamber of the heart or great vessel, is a rare congenital abnormality for which management has been previously described. However, there remains a paucity of literature on the treatment of sequelae, such as coronary artery aneurysm, in adults with acquired cardiac disease. We present an innovative surgical approach for the repair of an aneurysmal aortic root in a patient with a history of a coronary artery fistula repair and aortic valve replacement. The described operation integrates techniques of advanced aortic surgery with an interposition graft for the reimplantation of a massively dilated left main coronary artery. As life expectancy improves for patients with congenital cardiac abnormalities, the management of acquired cardiac disease must continue to evolve to appropriately treat this population.

## Introduction

Outcomes for patients with congenital heart disease (CHD) have improved significantly over time, with continued advancements in diagnostic modalities, surgical technique, and pediatric cardiology [1-4]. With decreased mortality, life expectancy has increased for this population. The median age of patients with CHD has risen from 11 years in 1985 to 25 years in 2010, with adults comprising two-thirds of the CHD population [5]. As these patients age, they face the challenges of heart failure, arrhythmias, and the need for further surgical interventions [6,7]. Subsequently, adult cardiac surgeons may frequently encounter these patients for the treatment of their disease. The unique history and anatomy of patients with CHD necessitate novel management strategies beyond standard surgical techniques.

Coronary artery fistulae (CAF), a rare congenital cardiac abnormality, are frequently surgically corrected due to a significant risk of eventual complications [8]. Previously manipulated coronary arteries may, over time, become ectatic or aneurysmal, requiring special care in future surgical interventions. This exacerbates the challenges of reoperative cardiac surgery, including disrupted tissue planes and adhesions from prior surgery [9,10]. There is a scarcity of literature detailing the surgical management of acquired cardiac disease for patients with previous CAF repair. We present a case detailing the successful operative management of an acquired aortic root aneurysm in a patient with multiple previous sternotomies and a history of left anterior descending (LAD) artery to right ventricular fistulae. The described techniques may be applied in future cases for patients with a similarly complicated surgical history.

## Case presentation

Our patient is a 53-year-old woman with a past surgical history of transventricular repair of multiple coronary artery fistulae between her LAD and right ventricle in her adolescent years, as well as redo sternotomy and mechanical aortic valve replacement for severe aortic insufficiency nine years later.

**Figure 1:**
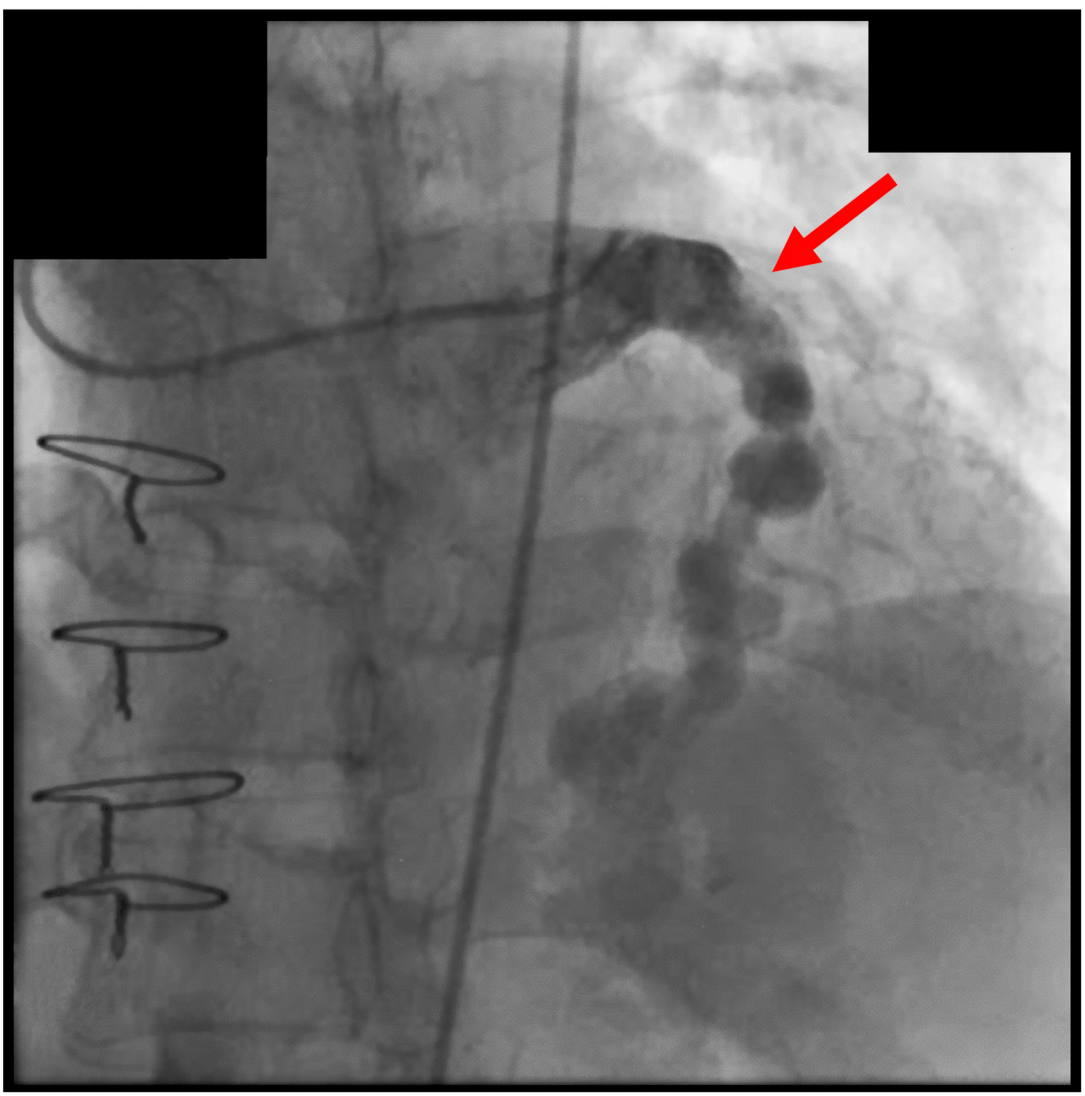
Preoperative cardiac catheterization demonstrating aneurysmal left anterior descending artery (red arrow) and fistulous connections within the interventricular septum.

The patient subsequently developed an aneurysm of the ascending aorta and proximal arch, up to 6.0 cm with 1 cm of growth during the prior year. She was referred for cardiac surgery to discuss operative management. Cardiac catheterization demonstrated an aneurysmal LAD with mural calcification, long segment ectasia, and multiple patent fistulous vessels within the interventricular septum (Figure 1). Her medical history was otherwise insignificant and without additional known comorbidities. She was highly active and expressed dissatisfaction with the lifestyle limitations of continued warfarin therapy. She was counseled regarding the risks and provided informed consent for redo sternotomy, aortic root replacement with a bioprosthetic valve conduit, ascending aortic repair, and arch replacement.

Based on experience with minimally invasive cardiac surgery and complex aortic reconstruction, modifications from standard surgical techniques were employed. Prior to the skin incision, a percutaneous pulmonary artery (PA) vent and a coronary sinus (CS) cardioplegia catheter were placed by the anesthesiologist via the right internal jugular vein. After standard preparation and draping for redo sternotomy, the innominate artery was identified and isolated above the sternal notch. Of unfractionated heparin, 5000 IU was administered, and a 10 mm woven double velour polyester graft was anastomosed to the innominate artery using a sidebiting clamp and 5-0 monofilament suture. The sternum was then divided without incident. The complete mobilization of the entire cardiac mass was avoided to reduce operative risk and allow focus to be placed exclusively on the exposure of the aortic root and arch. As expected, the root was densely adherent to surrounding structures, including the right atrium. When further dissection was deemed unsafe, a 25 mm percutaneous right atrial cannula was placed via the right femoral vein with transesophageal echocardiogram (TEE) guidance. Cardiopulmonary bypass and systemic cooling to 28 degrees centigrade were initiated. The ascending aorta and arch were then further mobilized in a safe and controlled manner.

An aortic cross-clamp was applied, and prompt cardiac arrest was achieved with retrograde del Nido cardioplegia. Maintenance cardioplegia and cold blood were given at predetermined intervals via the minimally invasive CS catheter. The ascending aorta was resected down to the sinotubular junction. A right coronary button was fashioned, and the noncoronary sinus was resected with cautery and sharp dissection. The previously placed mechanical valve was excised. The left coronary ostium was measured at 16 mm, with very fragile and attenuated tissue. A button encompassing the entire left coronary sinus was fashioned. The proximal left main coronary was calcified and aneurysmal and appeared inflamed. With some difficulty, the button was carefully mobilized.

**Figure 2:**
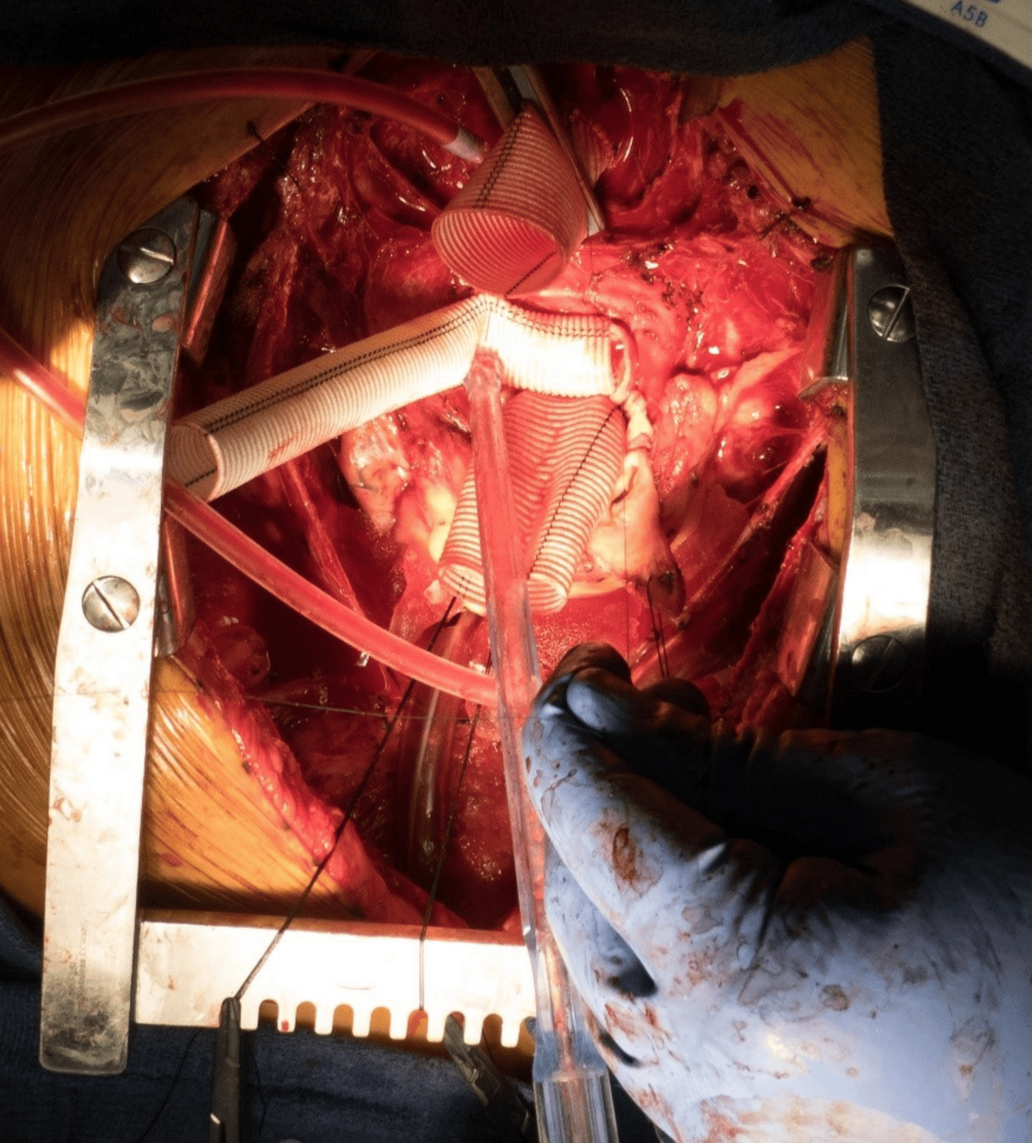
Intraoperative photograph demonstrating 16 mm woven polyester graft.

A custom-built bioprosthetic valved conduit was placed (23 mm Magna Ease valve {Edwards Lifesciences, Irvine, CA} and 26 mm Hemashield Dacron graft {Meadox Medicals, Inc., Oakland, NJ}) using interrupted pledgeted 2-0 Ethibond (Ethicon, Raritan, NJ) mattress sutures. The left coronary main button was of insufficient length to reach the root graft without tension on the friable, dilated sinus tissue. Further mobilization would place the coronary and surrounding structures at risk of irreparable damage. A short interposition graft was planned. The button was trimmed to the level of the dilated left main coronary, just short of the calcified portion. A 16 mm woven polyester graft was sutured to the left main orifice in an end-to-side fashion (Figure 2) using 5-0 polypropylene suture. The other end of the graft was then cut short on a bevel. An ostium was made in the root graft with electrocautery. Another end-to-side anastomosis was similarly performed. We then completed the remainder of the root replacement in the usual fashion.

At the aortic arch, a peninsula-style replacement was completed with a separate piece of 26 mm woven polyester graft. A graft-to-graft anastomosis was then performed. The right coronary button was reimplanted with the standard technique. The heart was de-aired, and the aortic cross-clamp was removed. After the restoration of adequate cardiac function, the patient was separated from cardiopulmonary bypass. Following the closure of the incision, the PA vent and CS catheter were removed.

**Figure 3:**
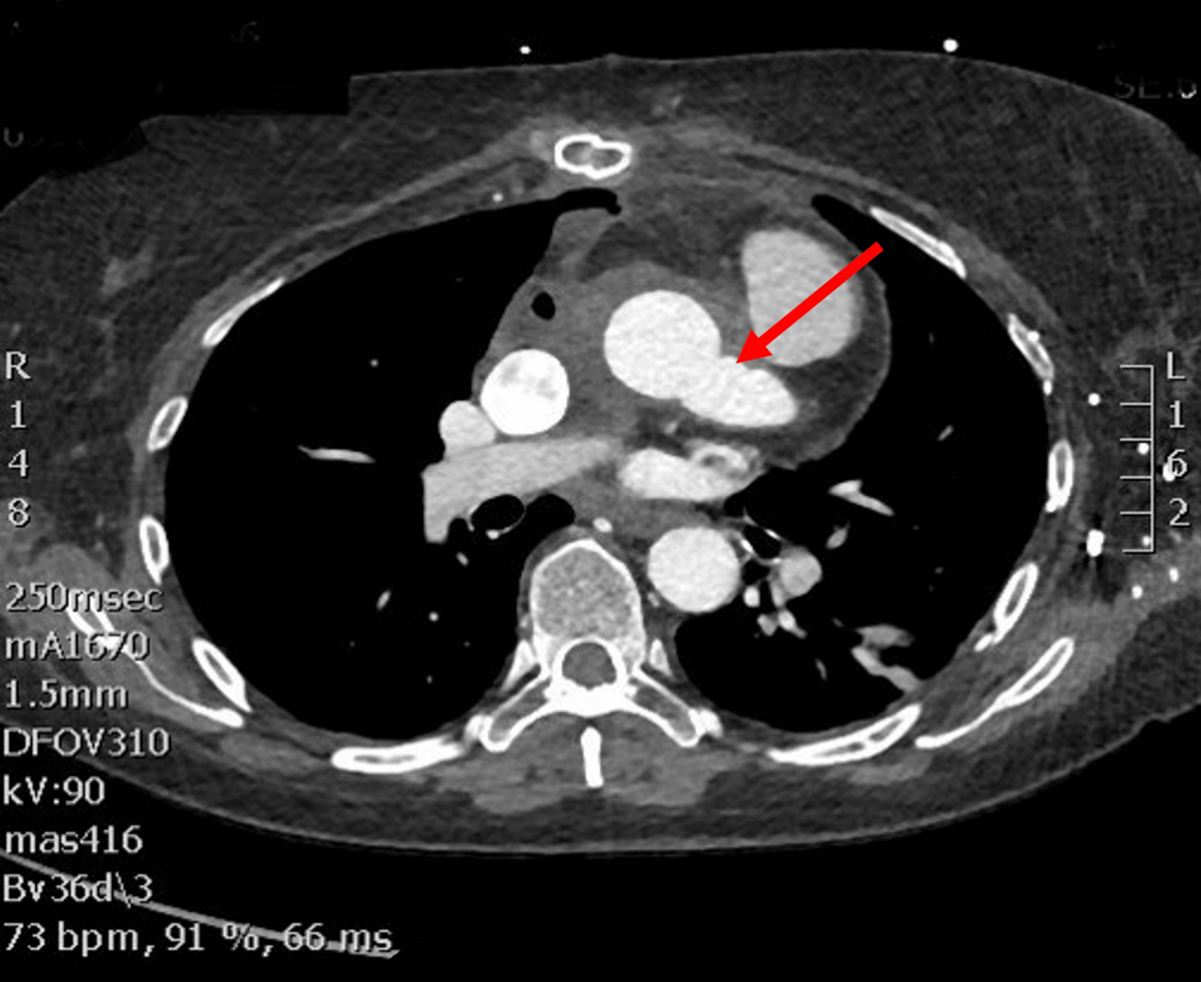
Postoperative computed tomography (CT) imaging demonstrating reconstructed left main coronary graft (red arrow).

The patient had an uneventful postoperative course. She was discharged from the hospital on postoperative day 7 and continued to recover well at home. Her computed tomography (CT) scan at the one-month follow-up demonstrated good flow in her reconstructed left main coronary graft without signs of kinking or twisting (Figure 3).

## Discussion

Coronary artery fistulae (CAF) are rare, occurring in one in 50,000 live births and comprising just 0.4% of all cardiac malformations. Although most frequently congenital lesions, CAF may also be iatrogenic or acquired via trauma or infection [8,9,11]. CAF most commonly drain into the right heart, as seen in this case. However, multiple fistulae, as present in our patient, are less common than a single fistula. Due to a high risk of eventual complications, including arrhythmia, myocardial ischemia, and valvular dysfunction, the surgical correction of CAF should be considered [8]. In patients with a history of CAF repair, recurrent or residual fistulae have been infrequently described and were without hemodynamic significance. The interval angiography of the native coronary artery in this population may demonstrate thrombosis or an ectatic and tortuous appearance, as seen in our patient. Results of CAF repair have been favorable but limited by a lack of long-term follow-up, up to a mean of 9.1 years in one study [10,12]. Long-term outcome research is necessary to further characterize the future clinical implications for these patients.

Due to the complexity of our patient's surgical history, an innovative approach to aortic root replacement was necessary. The dense adhesions present within the operative field were anticipated preoperatively. To limit hazardous dissection, techniques previously utilized in complex aortic and minimally invasive cardiac surgery were employed. In this case, the pulmonary artery vent and coronary sinus cardioplegia catheter were placed percutaneously via the right internal jugular vein, and cannulation was performed via the femoral vein and innominate artery.

In addition to the challenges of redo aortic surgery, the operation was further complicated by the presence of a dilated and friable left main coronary artery. The optimal management of a coronary artery aneurysm or ectasia (CAE) has not been clearly established in the literature. Studies have suggested potential benefit in the use of antiplatelet agents or anticoagulation in patients with concomitant acute coronary syndrome (ACS); however, additional research is indicated [13,14]. The surgical repair of CAE is associated with a high risk of complications and may include resection and the use of a synthetic or saphenous vein graft [13,15,16]. In our patient, a woven polyester interposition graft allowed for the successful reconstruction of the left main coronary artery.

## Conclusions

With the improved survival in congenital cardiac patients, adult cardiac surgeons will more frequently encounter previously repaired coronary anomalies. We describe the successful repair of a sinus of Valsalva aneurysm, with minimal manipulation of a fragile, dilated left main coronary artery. This patient's history and unique anatomy provided multiple challenges to a successful operation, including a redo sternotomy, the management of the proximal arch and the neurologic protection required, reoperative aortic root replacement, myocardial protection in the setting of a coronary to ventricular fistula, and the reimplantation of a highly abnormal left main coronary ostium. This challenging operation was simplified with the application of techniques learned from minimally invasive cardiac surgery and complex aortic arterial reconstruction procedures, such as the percutaneous pulmonary artery vent and coronary sinus cardioplegia catheter and the creation of synthetic interposition grafts. These techniques can be added to the surgeon's armamentarium to prepare for new surgical challenges, including the unique sequelae of previously repaired congenital cardiac abnormalities.
